# Diaphyseal femoral fatigue fracture associated with bisphosphonate therapy – 3 more cases

**DOI:** 10.3109/17453674.2010.539500

**Published:** 2011-02-10

**Authors:** Kaiya Osugi, Shigeki Miwa, Shinobu Marukawa, Kouhei Marukawa, Yoshiharu Kawaguchi, Shinichi Nakato

**Affiliations:** ^1^Department of Orthopaedic Surgery, Asahi General Hospital; ^2^Miwa Orthopedic Clinic; ^3^Marukawa Hospital; ^4^Department of Orthopedic Surgery, University of Toyama, Japan

## Case 1

**Figure 1.**
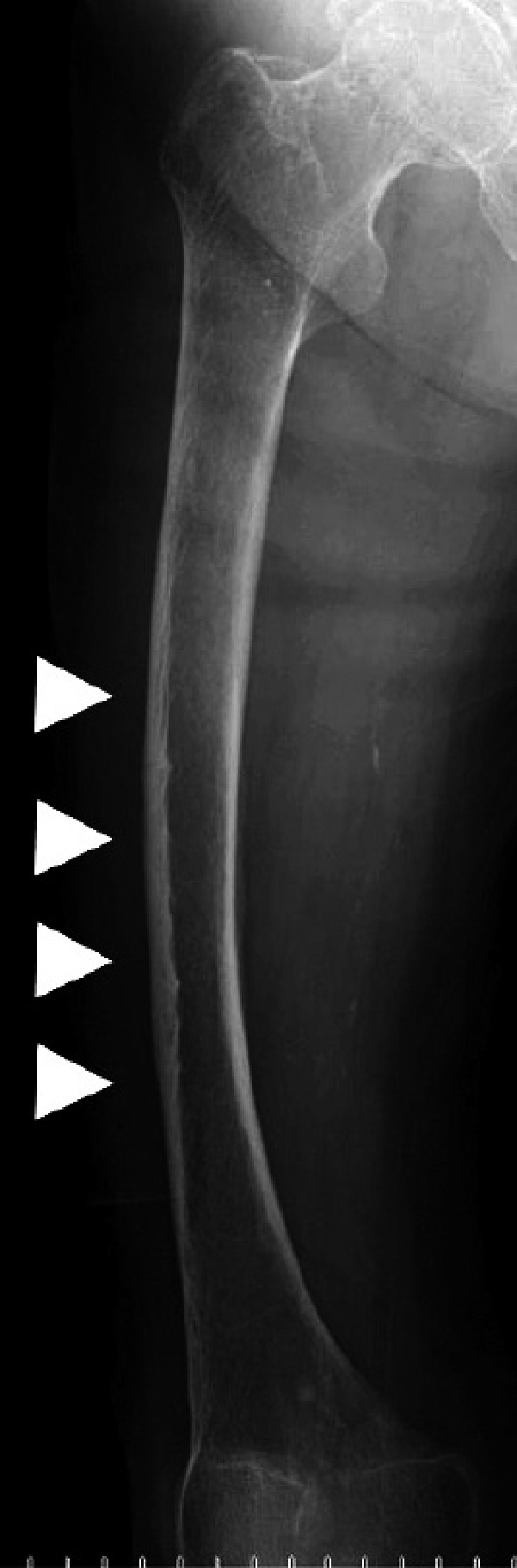
Case 1. The right femur showed lateral cortical thickening with a jagged pattern (arrow).

**Figure 2.**
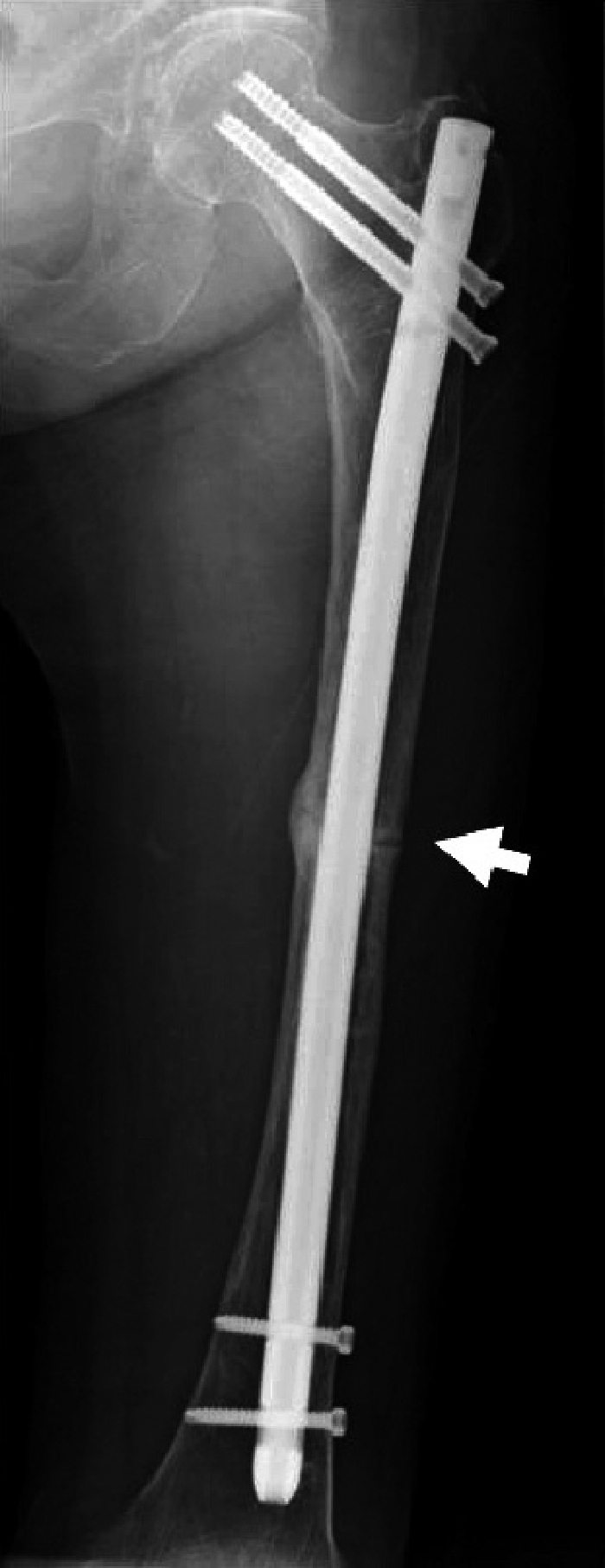
Case 1. Reduced fracture gap of the left femur (arrowheads) 1 year postoperatively.

In April 2006, a 77-year-old female fractured her left femur after falling. The cortex had a spike-shaped edge at the fracture site and also showed thickening (Figure 1). Intramedullary nailing was performed. The fracture gap remained open 1 year after surgery (Figure 2), but healing was confirmed by radiography 2 years after the injury. 2.5 years after the first injury, the patient fell while walking and contracted a diaphyseal transverse fracture of the right femur. The cortex had a spike-shaped edge at the fracture site.

The patient had been given ethidronate (4.8 g/12 weeks) from January 1999 to October 2001 because of postmenopausal osteoporosis. Thereafter, she was given alendronate (5 mg/day) for 7 years until the right femoral fracture had healed. Urine analysis conducted in February 2004 showed a free deoxypyridinoline/creatinine ratio (f-DPD/Cre) of 3.1, which is at the lower end of the normal range (2.8–7.6).

## Case 2

**Figure 3.**
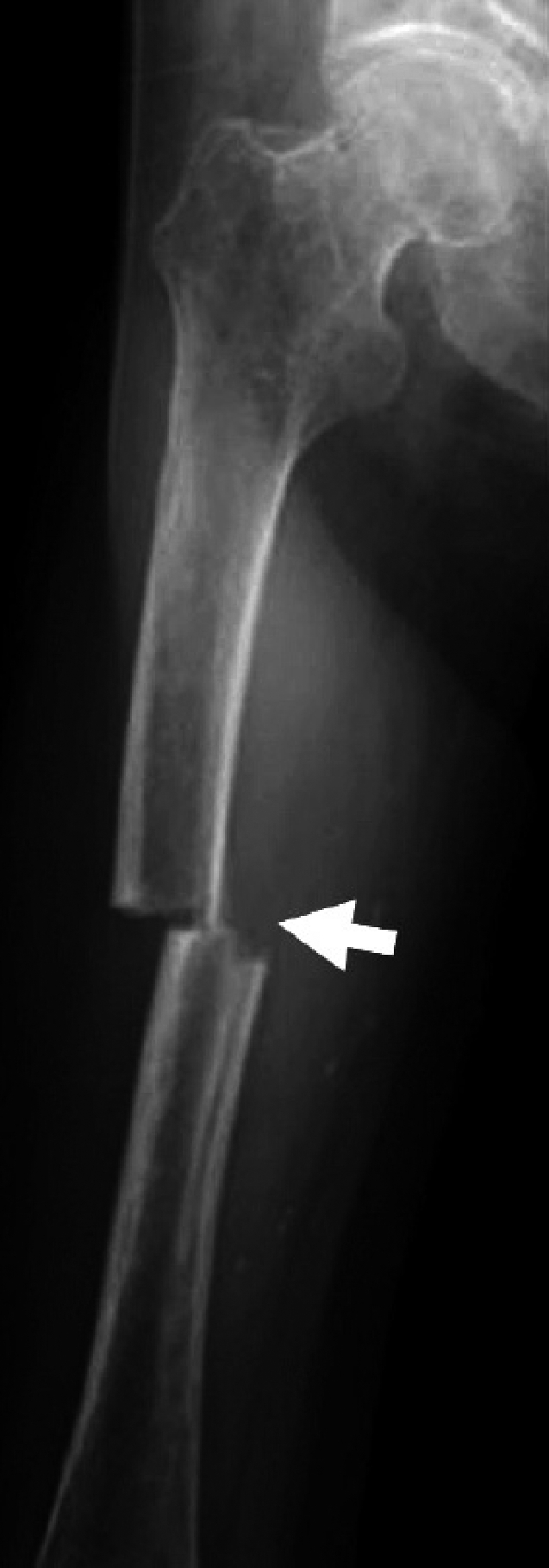
Case 2. Right transverse diaphyseal fracture with a medial spike (arrow).

**Figure 4.**
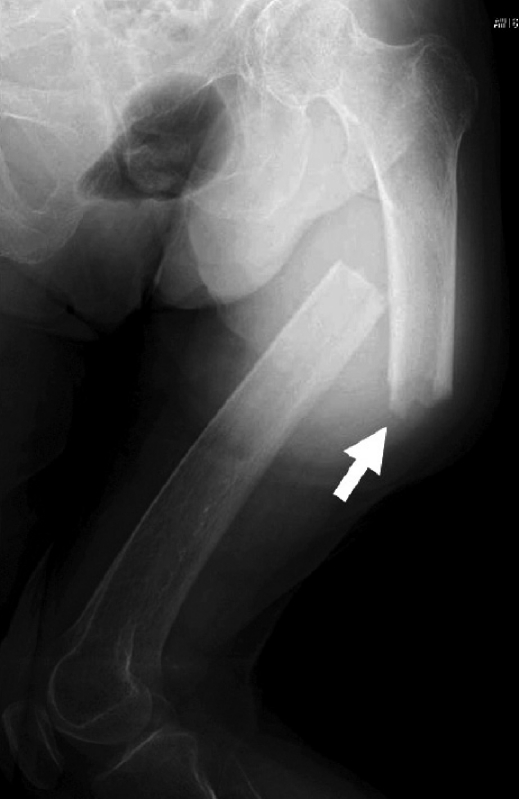
Case 2. The left femur showed cortical thickening with a jagged pattern (arrow).

In May 2009, a 78-year-old female felt that she was going to fall while walking, and contracted a diaphyseal transverse fracture. The cortex had a spike shaped-edge at the fracture site (Figure 3). The lateral cortex of the contralateral femur was also thickened (Figure 4). This patient had been administered risedronate (2.5 mg/day) for 2 years, from November 2002 until November 2004, because of postmenopausal osteoporosis. Thereafter, she was given alendronate (5 mg/day) until October 2008. In the next half-year period from October 2008, she was administered raloxifene (60 mg/day). Her tartrate-resistant acid phosphatase 5b (TRACP-5b) 2 days after the injury was 111 mU/dL, which is below the standard values for postmenopausal females (250–260).

## Case 3

In March 2004, a 70-year-old female fell and contracted a diaphyseal transverse fracture of her right femur with spike-shaped cortical thickening laterally. The lateral cortex of the left femur was also thickened. Union was confirmed by radiographs 6 months after intramedullary nailing. In September 2004, she felt that she was falling and experienced pain in her left femur. Radiographs showed an incomplete fracture, and she was advised to rest and support her leg. She was able to walk again, putting weight on the leg in December. In March 2005, she felt that she was about to fall and fractured the left femur. Again, the cortex presented a spike-shaped edge at the fracture. The patient had been administered risedronate (2.5 mg/day) from June 2002 through January 2005.

### Discussion

Suppression of bone resorption is the principal pharmacological activity of bisphosphonates. Bisphosphonates are therefore widely used in the treatment of osteoporosis. They reduce the risk of fractures (Kwek et al. 2008). However, the possibility of severely suppressed bone turnover (SSBT) under long-term administration has been cited (Black et al. 2006). In other words, repair of the microdamage to bones in daily life is inhibited by bone resorption suppression agents, and the cumulative effect of this microdamage is the mechanism behind vulnerability of the bone (Mashiba et al. 2000). Some characteristics of meta/diaphyseal stress fractures include (1) that they result from light trauma, (2) that they are simple and transverse, (3) that one side of the bone cortex shows a spike-shaped edge, (4) that there is thickening of the lateral cortex (Odvina et al. 2010), (5) that there is delayed union, and (6) that there is presentation of symptoms before the fracture (Ciarella et al. 2003). Points (1)–(4) were characteristics of all our cases. Furthermore, the fracture gap that persisted for a year in case 1 might be an example of (5). In addition, a reduction in bone resorption markers was confirmed in cases 1 and 2.

Since the first case of a fatigue bone fracture during treatment with alendronate, reported in 2005 by Odvina et al., there have been a number of such cases described in the literature (Black et al. 2006). Furthermore, there have been some rarer reports with risedronate (Ensrud et al. 2004, Goh et al. 2007). To our knowledge, however, there have been no previous reports regarding raloxifene. Here, in case 2, we report a fatigue bone fracture after 6 years of bisphosphonate treatment, followed by a switch to raloxifene. In case 3, a complete fracture occurred 2 months after cessation of bisphosphonate administration. This is the only report of fatigue bone fractures after transition from bisphosphonate to raloxifene treatment or after suspension of risedronate administration.

Several cases of fatigue fractures of the femur in patients on long-term bisphosphonate treatment were reported by Aspenberg (2009). Almost half of the patients had bilateral fractures and a quarter of them took corticosteroids. On the other hand, Schilcher and Aspenberg (2009) reported that the incidence density of stress fractures associated with bisphosphonate use was low and acceptable, considering that bisphosphonate treatment is likely to reduce the incidence density of any fracture. Black et al. (2010) concluded that the risk of fracture of the subtrochanteric or diaphyseal femur associated with bisphosphonate use was very low.
